# Next generation sequencing and tumor mutation profiling: are we ready for routine use in the oncology clinic?

**DOI:** 10.1186/s12916-014-0140-3

**Published:** 2014-08-12

**Authors:** Debu Tripathy, Kathleen Harnden, Kimberly Blackwell, Mark Robson

**Affiliations:** University of Southern California, Keck School of Medicine, USC/Norris Comprehensive Cancer Center, 1441 Eastlake Avenue, NTT-3429, Los Angeles, CA 90033 USA; Duke Cancer Institute, Duke University Medical Center, 2301 Erwin Road, Durham, NC 27710 USA; Memorial Sloan Kettering Cancer Center, Memorial Hospital, 1275 York Avenue, New York, NY 10065 USA; Weill Cornell Medical College, 1275 York Ave, New York, NY 10065 USA

**Keywords:** Breast cancer, Next-generation sequencing, Oncology

## Abstract

Next generation sequencing (NGS) coupled with sophisticated bioinformatics tools yields an unprecedented amount of information regarding tumor genetics, with the potential to reveal insights into tumor behavior. NGS and other multiplex genomic assays are rapidly spilling from the laboratory into the clinic through numerous commercial and academic entities. This raises the important question as to whether we are ready to use these data in clinical decision-making. While genetic lesions are clearly targeted by a new generation of biological cancer therapies, and certain regulatory approvals are actually coupled to single gene assays, we still do not know if the vast information on other genomic alterations is worth the added cost, or even worse, the inappropriate and unproven assignment of patients to treatment with an unapproved drug carrying potentially serious side effects. On the other hand, the trend toward a precision medicine pathway is clearly accelerating, and clinical trials validating pathway-driven personalized cancer therapeutics will be necessary in both the community and academic settings. Lower cost and wider availability of NGS now raises a debate over the merit of routine tumor genome-wide analysis.

## Introduction: Are we ready in the clinic?

**Figure 1:**
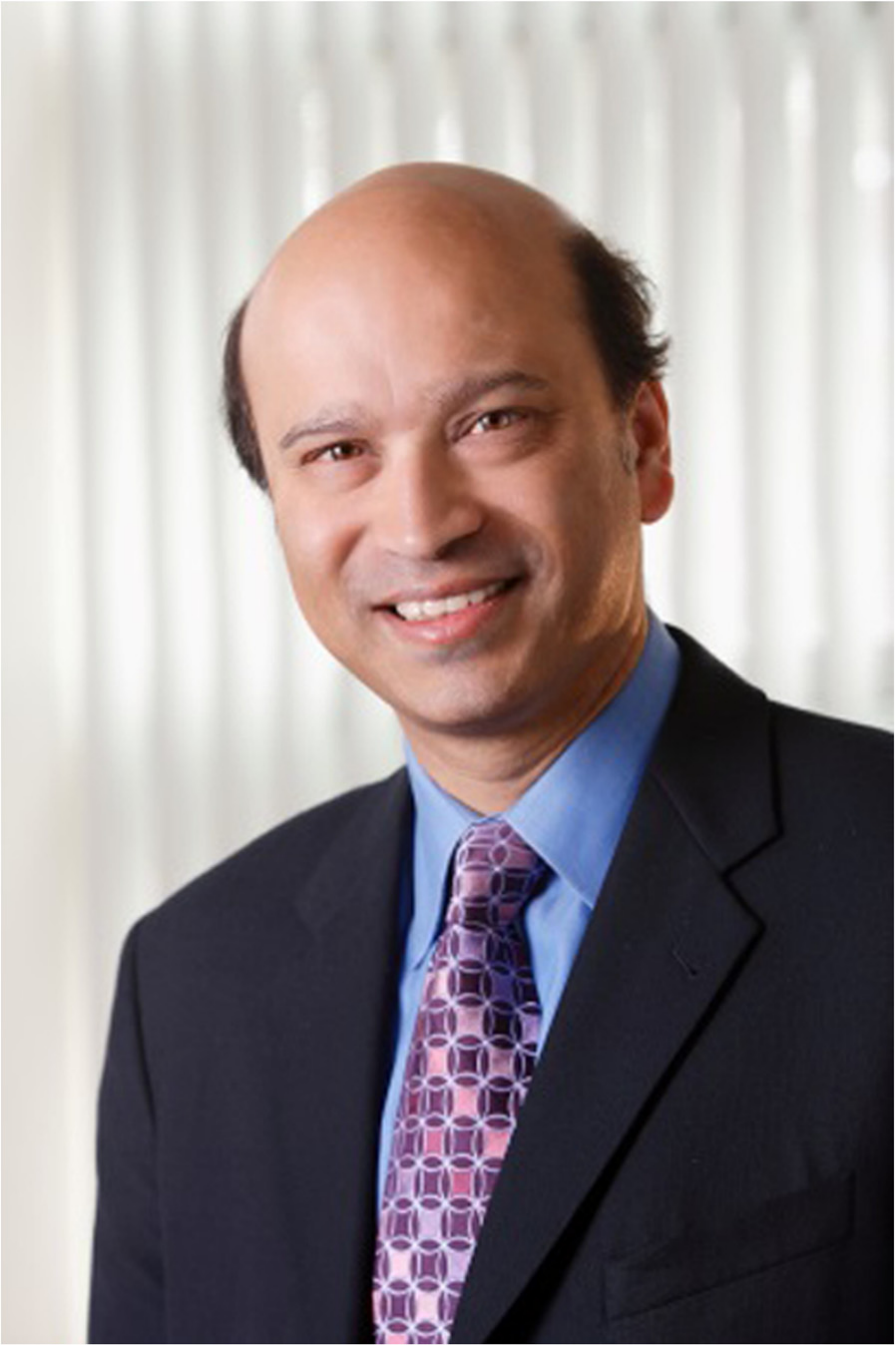
**Debu Tripathy, MD is Professor of Medicine and Co-Leader of the Women's Cancer Program at University of Southern California/Norris Comprehensive Cancer Center and holds the Priscilla and Art Ulene Chair in Women’s Cancer.** His area of clinical research interest is novel therapeutics in breast cancer, specifically, growth factor receptor pathway targeting as well as biomarkers that predict sensitivity and resistance. He is also part of a trans-disciplinary breast specialty team dedicated to patient-centered and personalized approaches to care, with an emphasis on translational clinical trials.

**Debu Tripathy (Figure ****1****)**

As with most new technologies and treatments, molecular diagnostics are entering the oncology arena in phases. However, these phases are not fully evidence-based or scientifically driven – they are somewhat organic as physician and patient interest always seems to be a step or two ahead of the data. The concept of ‘precision medicine’, defined as therapy that is personalized to unique disease and host characteristics, has taken huge leaps in the cancer field with the advent of next generation sequencing (NGS) [1,2]. NGS encompasses several technologies that generate gene sequence as well as copy number alterations and translocation of numerous genes. However, as pointed out by Dr. Robson, the most evidence-based use of genomic-based therapy still relies on single gene analysis, such as *EGFR* mutations and *ALK* rearrangements for lung cancer, *BCR-ABL* translocation (both presence and transcript quantification) for chronic myelogenous leukemia (CML) and *HER2* amplification for breast cancer [3-6]. The advantages of NGS are primarily the brute force of sequencing just about all expressed genes depending on the details of the platform, as well as the great ‘depth’, that is the number of ‘reads’ that allows for the detection of mutations that may be seen in only a fraction of cells (but are potentially the most dangerous ones). A disadvantage is the level of noise – mutations that may not be ‘drivers’ or may not be ‘actionable’, in that they do not clearly lend themselves to specific effective therapies. Several approaches have been devised to distinguish random (or ‘passenger’) mutations from true drivers, including direct testing of the genetic lesions in preclinical models or bioinformatics approaches that model the effects from the mutation sites and downstream biological pathways. We can also assume that more frequent mutations are those selected above the much rarer random ones due to their evolutionary selection as drivers of growth (or drug resistance in the case of those that may arise after treatment). Even mutations that can be biologically confirmed as mediators of malignancy and metastasis phenotypes and for which active drugs exist may not be clinically useful. This is evidenced by the successful targeting of *BRAF* mutation (V600E)-associated melanoma with BRAF and downstream MEK inhibitors, but the lack of efficacy in BRAF-mutated colorectal cancer, perhaps due to more genomic alterations and resultant bypass pathways [7,8].

NGS can generate results quickly since millions of pieces of small pieces of DNA are read in parallel. But tiling, or aligning the reads to generate interpretable results, requires complex bioinformatics support and a significantly longer time. For many tumor types, NGS has no proven benefit, although the theoretical advantage is that it may identify a clinical trial or even drug approved for a different cancer type that MIGHT be helpful. Accordingly, for today’s clinical practice, single gene assays suffice. However, as NGS become cheaper, it may be a simpler way to perform diagnostics on the small number of tumors in which several mutations, translocations or deletions are of proven benefit in decision-making, such as lung cancer or hematological malignancies. It also requires less tissue than multiple single gene tests. Also, customized panels for more commonly mutated genes in a specific cancer are becoming available that may offer focused multi-gene testing at a lower cost and yielding fewer ‘uninterpretable or unactionable’ findings.

NGS can also be applied to germline testing to detect heritable cancer susceptibility gene mutations and variants. It can also be helpful in the interpretation of tumor NGS results by excluding inherited variants that may be mono-allelic in the tumor. For familial genetic testing, NGS or multi-gene panel testing is being increasingly used as sequencing costs drop and can be informative in the setting of strong family histories without mutations seen in more common predisposition genes. In one series of patients, with a family history of breast cancer but no mutation seen in *BRCA* 1 or 2, a 42-gene panel assay found additional findings in 15 of 141 patients (11%), most of whom had breast cancer, with germline mutations/variants seen in *ATM, BLM, CDH1, CDKN2A, MUTYH, MLH1, NBN, PRSS1* and *SLX4* genes [9]. This technology could therefore address the need to lower cost and expand the scope of gene susceptibility testing, but it also creates clinical dilemmas in the management of the carriers of these gene anomalies for whom we do not fully understand the natural history and cannot provide lifetime cancer risk estimates.

### So when should we be using NGS or multi-gene testing?

From the standpoint of clinical utility, the bar is quite high, and has clearly not been reached – that would require the demonstration that the actions based on the test actually result in clinical benefit compared to decisions made without its use. At this time, single gene assays meet that criteria, but not NGS. In fact, we have not even excluded harm – an inappropriate or harmful decision from acting on a result of NGS given the vast amount of information contained in the result and the paucity of knowledge for most of the ‘targets’ listed. As an example, a recent trial for patients with advanced breast cancer that performed array comparative genomic hybridization for copy number variation and gene sequencing (*AKT* and *PI3KCA* only) and then assigned them to a panel of drugs that were known or presumed to address the genetic lesions. Of 423 patients enrolled, biopsies were obtained in 297, with genomic analysis feasible in 283, genomic alterations identified in 195, ‘personalized’ therapy available for 55, and in 43 patients treated with 16 different targeted regimens and evaluable for response, only 4 had objective responses and 9 had stability of disease [10]. These results illustrate the prematurity of using broad-based molecular testing routinely, but do point to the promise that these numbers can significantly improve with more comprehensive analysis and more experimental drugs available. Also, disease types with more ‘druggable’ targets, such as lung cancer and hematological malignancies, could yield different results and several prospective consortia are testing this approach. Drs. Harnden and Blackwell appropriately emphasize that as more patients and their treating oncologists are able to access a growing number of mutation-guided clinical trials, NGS may be justifiable – ideally as a formal part of such trials.

The future of genomically targeted cancer therapy will not only include NGS, but other high throughput technologies such as RNA sequencing that provides quantitative gene expression as well as mutational status and can also analyze micro- and noncoding RNAs that modulate gene expression. Proteomic analysis can also provide functional information that lends itself to drug selection. Also, genomic analysis after treatment progression can be very productive as demonstrated with studies of imatinib-resistant CML harboring the difficult-to-treat T315 resistance-associated mutation in BCR-ABL and resistance-associated mutations in EGFR, such as T790M in lung cancer, where this knowledge has led to the development of new therapeutics (ponatinib approved for CML and several drugs in testing for EGFR-kinase inhibitor-refractory lung cancer) [11,12].

Importantly, a growing body of information regarding mutational analysis in large populations in the context of clinical outcome and response to therapies will assemble a more robust understanding of cancer genomics and systems biology that can elucidate critical vulnerabilities that can be tackled with specific targeted drugs, or more likely, combinations of drugs [13,14]. The availability of portals for patients and physicians to understand better the nature and consequences of genomic alterations, available therapies and open clinical trials (such as MyCancerGenome) [15] will greatly aid in clinical decisions and trial referral. Finally, large trials with numerous available targeted drugs are planned, including the NCI Match demonstration trial across all solid tumors and the MASTER protocol as a regulatory approval pathway for second-line squamous cell lung cancer treatment [16]. Altogether, this spells for a bright future for this still nascent field that will lead to comprehensive and precision molecular cancer therapeutics.

### Competing interests

The author declares that he has no competing interests.

**Figure 2:**
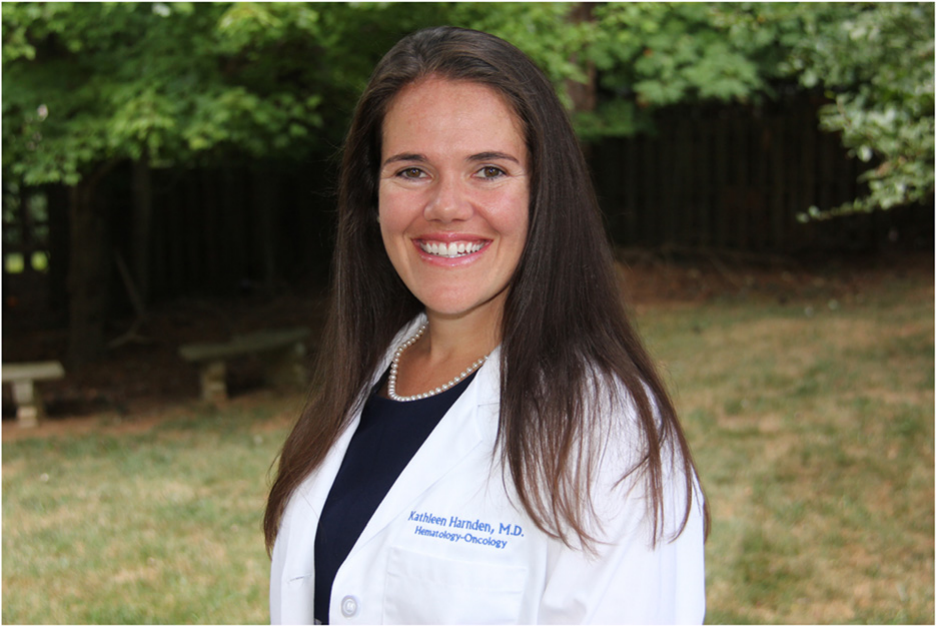
**Kathleen Harnden, MD is a senior fellow at Duke University Medical Center.** She graduated from Keck School of Medicine at University of Southern California and completed her internship and residency at Duke University Medical Center. Her research interests include breast cancer genetics and clinical trials in metastatic breast cancer.

**Figure 3:**
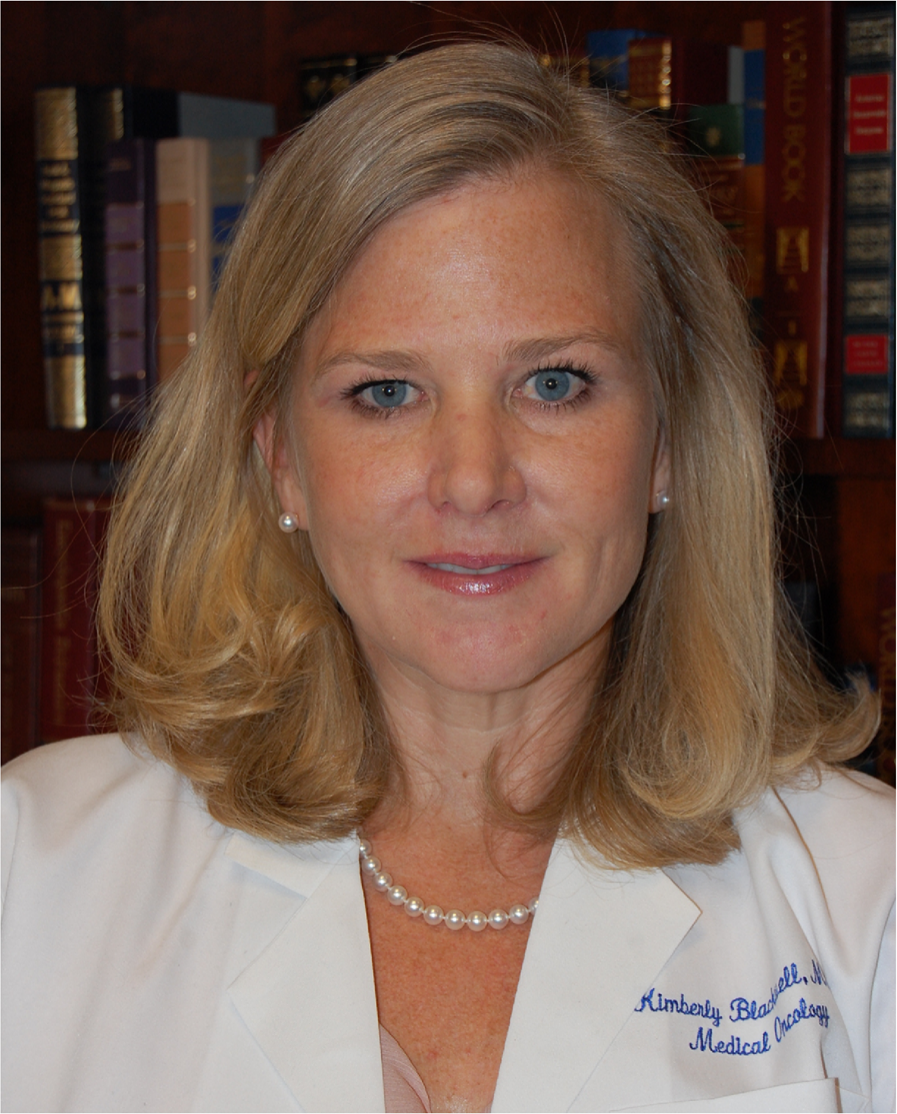
**Kimberly Blackwell, MD is professor of medicine and assistant professor of radiation oncology at Duke University Medical Center and director of the breast cancer program at the Duke Cancer Institute, where she oversees all basic and translational research programs involving breast cancer patients.** She has played a major role in two recently approved breast cancer drugs, lapatinib and T-DM1, both of which were studied in her laboratory and developed in trials in which she served as principal investigator.

## Tumor mutation profiling in breast cancer is ready for routine use in the clinic

**Kathleen Harnden and Kimberly Blackwell (Figures****2****and****3****)**

The foremost priority in cancer research is personalized treatment approaches offering the promise of more effective and better tolerated therapies. One major step towards offering personalized therapy is the use of genomic mutational analysis to help drive decision making in the clinic. Technologies such as NGS offer comprehensive analysis of specific mutations present in each patient’s breast cancer. The argument to include NGS in clinical decision making is based on several concepts including: 1) Actionable mutations are well described in the literature and targeted therapies are now available in the approved or trial setting; 2) Mutations might provide predictive capabilities for certain types of standard and experimental therapies and, therefore, offer an enrichment strategy in designing clinical trials; and 3) Mutations might offer prognostic and/or predictive information in certain types of breast cancer, such as HER2+ and triple negative breast cancer. We will now discuss each of these points in some detail.

There are many biologically meaningful and, therefore, actionable mutations described in cancer with currently available therapeutic options. These mutations are not confined to specific tissue origins. Instead, these mutations define subgroups of cancer in ways that we do not currently use clinically. The number of mutations with targeted therapies in breast cancer is ever increasing and currently includes genes such as poly (ADP-ribose) polymerase (PARP) (PARP inhibitors), p53 (vaccine therapy, gene therapy, Wee-1 inhibitors, Kevetrin), estrogen receptor alpha (ESR1) (alternative endocrine therapies), JAK1 (JAK1 inhibitors), and mTOR (mTOR inhibitors) [17]. There are also new actionable mutations on the horizon that are not well described in breast cancer with developing targeted therapies, including mutated genes such as dynein (hsp90 inhibitors, HDAC inhibitors), MST1 (anti-MST1 receptor antibodies), ROS-1 (inhibitors), HGF (antibodies against c-met, c-met inhibitors), and ALDH8A1 (disulfiram). NGS empowers providers to identify and exploit driving mutations in breast cancer biology and to employ new real time therapeutic options in the clinic.

Additionally, NGS is ready for routine use in the oncology clinic as it could create a redesign in the way patients are enrolled in clinical trials. NGS identifies meaningful mutations, allowing improved and more rigorous enrollment in clinical trials. These actionable mutations allow providers to transform standard treatment plans to individualized approaches and match patients to clinical trials. As NGS becomes more readily available and affordable, it should allow for better enrichment strategies for enrollment into clinical trials. To use NGS to enrich trial populations should be a very attractive idea to both study sponsors and patients.

An example of using NGS for trial enrichment is found in the SAFIR01 study. In a prospective trial of more than 400 breast cancer patients, André *et al*. found a targetable genomic alteration in 46% of patients [10]. Of these patients, 25% had treatment driven by genomics and 28% of the patients who were treated with genomic-driven therapy had an objective response or stable disease for up to 10 months. In this heavily pre-treated population of patients, the ability to find actionable targets in nearly half of the patients and provide benefit to the nearly 1/3 who received the treatment is clinically meaningful. Additionally, clinical trials of targeted agents in which patients would have been deemed ineligible using standard testing methods are now being designed to include NGS [18]. Patients with activating mutations of HER2 who were considered ineligible for standard HER2 based therapy now have new clinical trial options and therapeutic options based on sequencing results [18].

Another example of how NGS can enrich trial populations is found in the NCI MATCH (Molecular Analysis for Therapy Choice) study [16]. In this study, 1,000 patients with common and rare tumors will be enrolled for NGS at the time of progression to elucidate resistance mechanisms and to choose single agent or combination targeted therapy for these patients. There are currently 20 ‘arms’ of the study and more than 40 targeted agents pledged for use. The techniques, reliability and workflow are currently being optimized to streamline the NGS data for real time clinical impact.

A third reason that NGS is ready for the clinic is that it could better define prognosis (prognostic value) or potential compounds that have a higher likelihood of benefiting patients (predictive value). Each specific oncogenic event provides an opportunity for targeted therapy. If we can identify a molecular profile associated with greater therapeutic benefit we can prospectively select patients accordingly. Similar to the increased benefit of platinum therapy in metastatic triple-negative breast cancer in BRCA 1/2 positive patients [19] and a poorer prognosis in docetaxel and trastuzumab +/- pertuzumab therapy in HER2 amplified metastatic breast cancer in patients with a PIK3CA mutation [20], NGS can provide a foundation on which to maximize clinical benefit of highly targeted therapies based on tumor mutational signatures.

Finally, NGS could influence decision making in the clinic in determining prognosis in certain subtypes of metastatic breast cancer patients. O’Shaughnessy *et al*. found that extraordinary responders to lapatinib with trastuzumab primary-refractory inflammatory breast cancer share a common cancer genotype [21]. These cancer genotypes and their response phenotype can be elucidated for innumerable therapy regimens and used to make predictably beneficial treatment decisions in the clinic [22-25]. We presented data at the 2013 San Antonio Breast Cancer Symposium identifying gene mutations associated with progression free survival (PFS) in metastatic breast cancer [26]. Patients with WNK1 mutations had a median PFS of 1.4 months compared to 7.3 months (*P* = 0.03) in those with the wild type gene. Conversely, patients with mutated MST1 had improved PFS of 11.4 months compared to 6.2 months (*P* = 0.04) with the wild type gene. Likewise, several gene mutations conferred differences in overall survival. This prognostic information, obtained using NGS, could become incredibly valuable in counseling patients and in selecting appropriate therapies.

Although many of the therapeutic implications of NGS involve clinical trial participation, NGS is ready for the clinic. Some clinicians would argue it already is in the clinic. Our focus now should turn to bringing this revolutionary, therapy-altering and prognostic technology to all patients in an efficient, affordable way. In order to achieve this goal, scientists, bioinformatics specialists, clinicians and patients will need to work together to bring NGS to its full capacity.

### Competing interests

Dr. Harnden has no competing interests. Dr. Blackwell’s Institution has received recent research funding from GSK, Bristol Meyer Squibb, Celgene and Genentech. Dr. Blackwell has served as a consultant to Novartis, Celgene and Genentech.

**Figure 4:**
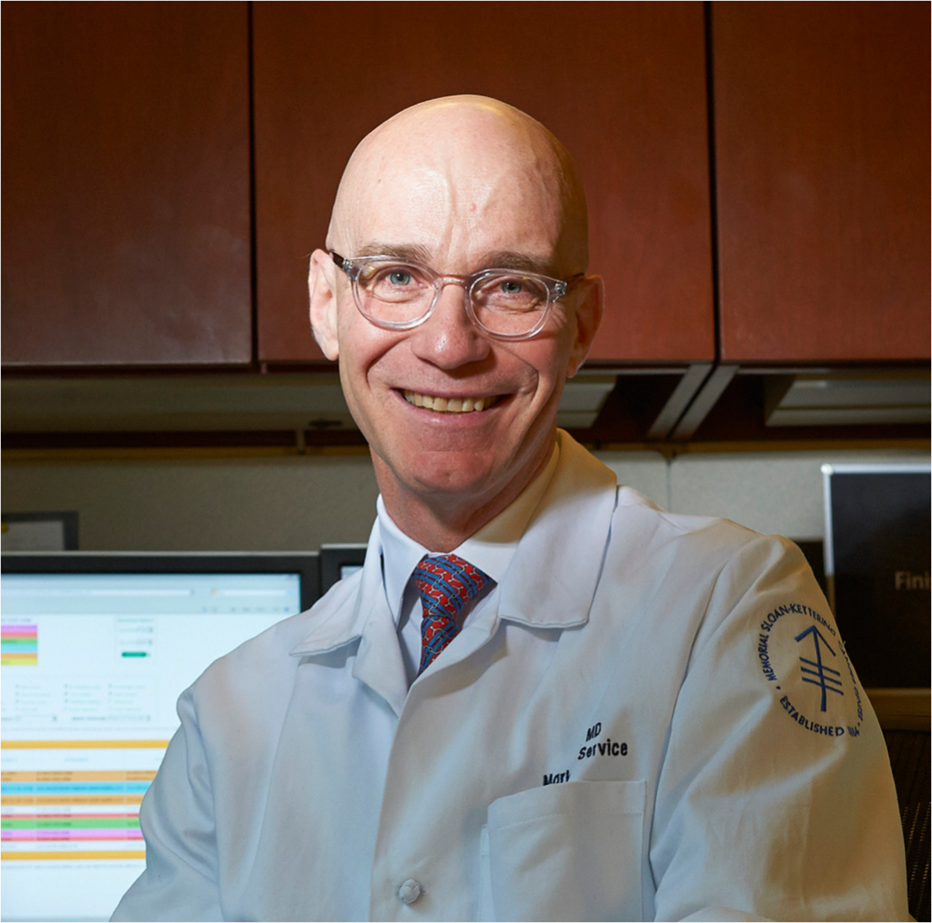
**Mark Robson, MD is an Attending Physician of the Clinical Genetics and Breast Medicine Services in the Department of Medicine at Memorial Sloan-Kettering Cancer Center.** His research is directed toward improving the integration of genetic information into the clinical management of women with breast cancer. He and his colleagues have conducted a number of studies examining outcomes in women with hereditary breast cancer to better define the risks and benefits of treatments such as breast conserving therapy and adjuvant chemotherapy in this group. He is currently conducting studies to evaluate the impact of intensive screening or surgical prevention upon women's quality of life and to develop new screening tools for breast cancer, such as serum peptide profiling.

## Tumor mutation profiling is not ready for routine use beyond the research setting

**Mark Robson (Figure ****4****)**

Cancer is a genetic disease, in the sense that the malignant phenotype arises, in large part, as a result of mutations in the DNA of the cancer cell. Massively parallel NGS offers the opportunity to define the pattern of genomic alterations in a patient’s cancer quickly and affordably. This capability could, in theory, fulfill the promise of ‘precision medicine’ and allow treatment tailored to the specific drivers of the individual patient’s disease. This promise has captured the imagination of oncology professionals, and a number of commercial and academic laboratories now offer tumor mutation profiling. Like any new technology, however, tumor mutation profiling with NGS is most appropriate for specific purposes. At this point, NGS is a tool for oncology research and does not yet have a place in routine clinical care.

There are three main ways that an oncologist could use information about the pattern of mutations in a patient’s cancer. First, he or she could use the information to select a treatment that has been approved by regulatory authority for use in the setting of a particular genetic change. Second, the oncologist could use the information to identify patient eligibility for clinical trials of new targeted agents. Finally, the oncologist could use the information to select a targeted treatment for ‘off-study’ and ‘off-label’ use. At this time, it is not necessary to employ NGS for the first purpose, and inappropriate to utilize NGS for the last. There may be a limited role for NGS for the second purpose, at least in academic medical centers with early development programs, but in most clinical situations, more directed testing is more appropriate.

There are increasing numbers of agents that are approved for the treatment of cancers harboring specific genomic alterations. The canonical example, of course, is the use of imatinib in the treatment of chronic myelogenous leukemia harboring the *BCR/ABL* rearrangement [27].A more recent example is the remarkable success of BRAF inhibitors in the treatment of malignant melanomas with *BRAF* V600E mutations [28]. Tumor mutation profiling can identify these mutations, of course, but the specific alterations appropriate for treatment with approved agents are well-defined, and there are usually specific companion diagnostic assays. There is no obvious benefit to using a NGS assay to identify an established mutation, and likely significant incremental cost. So, if there is a known target in a particular disease type, NGS profiling is not necessary.

At the other end of the spectrum is the hope that NGS may identify a therapeutic target that would not have been considered based on disease type, and that this knowledge will allow the oncologist to select an ‘off the shelf’ treatment for a patient that will be superior to a more traditional physician-selected therapy. While this is the application that is the most logical in the popular imagination, it is also the one that is the least supported by data. Even if one identifies a mutation that confers sensitivity in a particular disease, it may not predict response in another context. An example is the relative insensitivity of BRAF-mutant colon cancer to BRAF inhibition, despite the presence of the same mutation that confers sensitivity in melanoma [8]. The situation becomes even more complicated when one attempts to predict response based upon non-canonical alterations in genes that could be plausible targets for existing drugs. If the platform evaluates tumor DNA alone, any identified sequence change may, in fact, be germ line (inherited) in origin. The mere fact that a variation is rare does not argue for pathogenic relevance, since most normal human variation is common, and even protein-truncating mutations are seen in the constitutional DNA of apparently healthy people [29,30]. To identify sequence variants that are present in tumor only, a number of laboratories now sequence tumor and normal samples simultaneously, and ‘subtract’ the germ line sequence from the somatic sequence in order to identify those variants that are unique to the tumor. However, even somatic variants may not be causative. Such variants may not be functionally significant, and the process of determining causality is both complex and partly subjective despite the best efforts of a number of groups to standardize the process of curation [31]. Even if causative, the variant may not be directly related to the cancer process (‘passenger’ rather than driver’ mutation), in which case targeting the mutation will not have an effect on the growth of the tumor.

Even if a sequence variant is somatic, functional and related to the cancer process, targeting it may not be beneficial because it may not be present in the metastases that are threatening the patient. Genetic heterogeneity is an enormous challenge, both as a possible substrate for the development of resistance and as a source of variation between primary and metastatic sites (as well as between metastatic sites) [32]. Inadequate sampling may limit the ability to delineate the actual genomic changes that are most relevant to treatment.

Even if the variant is relevant in light of all of these considerations, it may not be targetable. There may not be an ‘off the shelf’ drug that can attack the vulnerability or tumor context may limit response (as discussed previously for BRAF inhibitors). Even if a drug is available, resistance to single agent targeted treatment often evolves quickly in solid tumors [33], and untested combinations of targeted agents are extremely unwise without careful delineation of toxicities in properly conducted clinical trials. Finally, even if a particular genomic alteration is relevant and targetable, the genomically-directed treatment may not be superior to conventional therapy, and foregoing a proven approach in favor of an unapproved targeted treatment may lead to inferior outcomes. The comparison of genomically-directed treatment to conventional therapy requires carefully designed, innovative clinical trials such as the recently announced Friends of Cancer Research-NCI Lung Cancer Master protocol [16,34].

While tumor mutation profiling does not yet have a role in determining treatment off-study, it may be a useful means of identifying patients who are candidates for studies of new genomically-directed therapies. At academic centers with extensive portfolios of new agents, it may be productive to conduct routine tumor profiling on patients with advanced disease in order to ‘pre-form’ cohorts of characterized patients who can be expeditiously directed towards appropriate studies when the time is right. Outside of the academic setting, however, patients may have limited geographic and financial access to trials, and it is not clear that it is helpful to them to learn about a study that they cannot travel to or afford. An alternative would be to conduct limited genomic characterization to determine eligibility for studies that are practical for the specific circumstance, rather than broad profiling.

In summary, then, somatic mutation profiling by NGS is not necessary for deployment of approved genomically-directed treatments and is not yet at the point where it can be used to direct off-protocol treatment. Profiling may be useful as a screening tool to determine trial eligibility but, for most patients, it may be more practical to employ a more limited approach to determine eligibility for specific studies of interest to the particular individual, given their clinical circumstances.

### Competing interests

The author declares that he has no competing interests
